# Separation and Purification of Biogenic 1,3-Propanediol from Fermented Glycerol through Flocculation and Strong Acidic Ion-Exchange Resin

**DOI:** 10.3390/biom10121601

**Published:** 2020-11-26

**Authors:** Laura Mitrea, Loredana Florina Leopold, Cosmina Bouari, Dan Cristian Vodnar

**Affiliations:** 1 Institute of Life Sciences, University of Agricultural Sciences and Veterinary Medicine Cluj-Napoca, Calea Mănăștur 3–5, 400372 Cluj-Napoca, Romania; laura.mitrea@usamvcluj.ro; 2 Faculty of Food Science and Technology, University of Agricultural Sciences and Veterinary Medicine Cluj-Napoca, Calea Mănăştur 3–5, 400372 Cluj-Napoca, Romania; loredana.leopold@usamvcluj.ro; 3 Faculty of Veterinary Medicine, University of Agricultural Sciences and Veterinary Medicine Cluj-Napoca, Calea Mănăștur 3–5, 400372 Cluj-Napoca, Romania

**Keywords:** 1,3-propanediol, cationic resin, flocculation, *Klebsiella pneumoniae*, purification

## Abstract

In the present work, was investigated the separation and purification procedure of the biogenic 1,3-propanediol (1,3-PD), which is a well-known valuable compound in terms of bio-based plastic materials development. The biogenic 1,3-PD was obtained as a major metabolite through the glycerol fermentation by *Klebsiella pneumoniae* DSMZ 2026 and was subjected to separation and purification processes. A strong acidic ion exchange resin in H^+^ form was used for 1,3-PD purification from the aqueous solution previously obtained by broth flocculation. The eluent volume was investigated considering the removal of the secondary metabolites such as organic acids (acetic, citric, lactic, and succinic acids) and 2,3-butanediol (2,3-BD), and unconsumed glycerol. It was observed that a volume of 84 mL of ethanol 75% loaded with a flow rate of 7 mL/min completely remove the secondary metabolites from 10 mL of concentrated fermented broth, and pure biogenic 1,3-PD was recovered in 128 mL of the eluent.

## 1. Introduction

Residual biomass, such as raw glycerol derived from biodiesel manufacturing, represents a sustainable source of nutrients for the biological catalysts that can convert it into valuable metabolites [1,2,3,4], which can further represent base materials for the production of eco-friendly plastic materials, for example, polytrimethylene-terephthalate (PTT) [5,6].

Even though 1,3-PD biosynthesis from renewable biomass is very attractive from the environmental and economic point-of-view, unlike its production through chemical synthesis, the high purity of biogenic 1,3-PD needed for further industrial usages constitutes a bottleneck of the entire process [7,8]. Mainly because the biogenic 1,3-PD is generated in low amounts in the cultivation media, the removal of other constituents like cells, proteins, salts, organic acids, and alcohols increases the total cost of the purification process for the targeted compound [9,10].

The separation of 1,3-PD from the cultivation media usually implies at least two steps. Commonly, the coarse fragments like cells and cell debris are removed by centrifugation, filtration, or microfiltration from the fermented broth [11]. The protein fractions, macromolecular pigments, and inorganic salts can be successfully separated from the cultivation media by nontoxic methods such as flocculation with a mixture of chitosan or polyacrylamide [12,13,14], activated charcoal [15,16], and kieselguhr (diatomite earth) [8,17,18].

Many purification methods have been investigated in the last two decades [8,9,11,19,20,21,22,23] for an increased yield of 1,3-PD, and ion exchange resins are considered as sustainable materials for 1,3-PD recovery due to their ability to be regenerated periodically [24,25,26]. In the case of 1,3-PD, divinylbenzene-based cation-exchange resins in different forms (H^+^, Ca^2+^, Ag^+^, Na^+^, Pb^2+^, Zn^2+^, Li^+^, Co^2+^, Cu^2+^) are suitable for the increased recovery of the compound. Still, resins in H^+^ and Ca^2+^ have been proven to have higher efficiency in 1,3-PD purification towards other forms of resins [8,20,27].

Besides ion-exchange resins, other methods of 1,3-PD purification have been reported, but many of them have certain drawbacks especially due to their high cost of the downstream process, or because of their increased grade of toxicity [9]. Wu et al. [28] used electrodialysis through bipolar membranes to desalinate the fermentation broth, to recover the salts, and to convert them into high-added-value by-products [28]. In this case, the membrane reuse limits its working life and increases the cost of the purification process. Hao et al. applied a reactive extraction of 1,3-PD from a dilute aqueous solution by using aldehydes (propionaldehyde, butyraldehyde, and isobutyraldehyde). Aldehydes that were found in excess replaced 1,3-dioxanes, from where 1,3-PD were further extracted by using reactive distillation equipment [7]. The reactive extraction of 1,3-PD utilizing acetaldehyde instead was applied by Malinowski [29], who obtained 2-methyl-1,3-dioxane which was further subjected to extraction by using organic solvents such as *o*-xylene, toluene, or ethylbenzene that were converted back to 1,3-PD by hydrolyzation [29]. In reactive extraction scenarios, the purification processes have some disadvantages because the aldehydes used as both extractant and reactant have a certain degree of toxicity, and at the same time, they can react with other alcohols from the fermentation broth like ethanol, 2,3-BD, or glycerol [30]. In a study conducted by Waszak et al., nanofiltration was applied as a cheaper method to separate 1,3-PD from fermented glycerol by *Citrobacter freundii* [31]. In their investigation, the fermented broth consisting of up to 5 g/L 1,3-PD was subjected to nanofiltration to extract the interest compound. The authors used an active membrane of 150 cm^2^, model NF270 from Dow FilmTec (Minneapolis, MN, USA). The membrane retained the major part of the cultivation media components like salts (NH_4_^+^, SO_4_^−^, K^+^, Na^+^, PO_4_^3−^), carboxylic acids (lactic, succinic, acetic acids), or glycerol, and separated 1,3-PD. In addition, there was also the possibility to reuse the membrane for a second filtration to diminish the total cost of the operation [31].

A particular aspect that makes the 1,3-PD separation process more complicated is related to its high hydrophilic characteristics, and its close boiling point to 2,3-BD and glycerol. In this context, the recovery of 1,3-PD from a microbial fermentation media would consume a large amount of energy and would make up over 50% of the total production costs [9,22]. In addition, the elevated polarity of 1,3-PD makes its extraction difficult from the aqueous system. Müller and Górak [32] investigated the possibility of 1,3-PD separation by applying two-phase aqueous systems. The authors of this study tested various ionic liquids starting with 1-butyl-3-methylimidazolium trifluoromethansulfonate as the initial ionic liquid, and different anions and cations like dicyanamide, thiocyanate, methysulfate (as anions), and 1-butyl-3-methylmorpholinium, 1-butyl-3-methylpyrrolidinium, 1-ethyl-3-methylimidazolium, and 1-methoxyethyl-3-methylimidazolium (as cations). The results of this study highlight that all of the investigated two-phase systems were appropriate for the extraction of 1,3-PD. Moreover, the 1,3-PD distribution coefficient is closely correlated with the polarity or hydrogen-bond accepting strength of the cation and anion [32].

In the present work, the capacity of acidic ion-exchange resin in H^+^ form (Amberlite IR-120H) to purify the biogenic 1,3-PD from the fermentation broth, after it was subjected to flocculation with chitosan, charcoal, and kieselguhr, was investigated.

## 2. Materials and Methods

### 2.1. Reagents

All reagents used for the present work were analytically graded. 1,3-PD (C_3_H_8_O_2_, purity 99%, d: 1.053 g/cm^3^, MW: 76.10 g/mol) was purchased from Alfa Aesar (Thermo Fisher Scientific GmbH, Kandel, Germany); 2,3-BD (C_4_H_10_O_2_, purity 98%, d: 1.002 g/cm^3^, MW: 90.12 g/mol) was provided by Sigma-Aldrich (Sigma-Aldrich Trading Co., Shanghai, China); glycerol (C_3_H_8_O_3_, purity 99%, d: 1.256 g/cm^3^, MW: 92.10 g/mol); organic acids (citric, succinic, lactic, and acetic acids), ethanol, and the components used for cultivation media were supplied by VWR Chemicals (VWR International GmbH, Langenfeld, Germany).

Calcinated kieselguhr (Celite Filter Cel) was acquired from Honeywell-Fluka (Honeywell Specialty Chemicals GmbH, Seelze, Germany). Chitosan (Poly-d-glucosamine) and cationic resin (Amberlite IR 120, hydrogen bond) were bought from Sigma-Aldrich (Sigma-Aldrich Trading Co., Shanghai, China).

The instrumentation used for the experimental part of the present work consisted of a bioreactor (Eppendorf, Hamburg, Germany), a centrifuge (Eppendorf, Hamburg, Germany), a magnetic stirrer plate (Ika Labortechnik, Staufen, Germany), a glass vacuum filtration unit (VWR International GmbH, Langenfeld, Germany), a rotary evaporator with vacuum (Heidolph Instruments, Schwabach, Germany), and an High Performance Liquid Chromatography with Refractive Index Detector system (HPLC-RID, Agilent 1200, Santa Clara, CA, USA).

### 2.2. Fermentation Process

The bacterial strain used for the fermentation of glycerol was *K. pneumoniae* DSMZ 2026, and it was purchased from the German Collection of Microorganisms and Cell Cultures (DSMZ, Braunschweig, Germany). The strain was cultivated in a 5-L Eppendorf bioreactor (model: BioFlo 320, one unit) filled with 2 L of culture broth (Eppendorf, Hamburg, Germany). The cultivation medium components and the fermentation conditions were described in our previous publications [33,34]. The fermented broth consisting of fully developed bacterial cells and metabolites was collected and subjected to separation and purification processes.

### 2.3. Broth Flocculation

The cell biomass from the fermentation broth was removed by high-speed centrifugation (10,000 RPM) for 10 min at 4 °C (centrifuge 5810R, Eppendorf, Hamburg, Germany). The supernatant was collected and stored at −20 °C for later use.

To remove the organic macromolecules, cells debris, and proteins, the fermentation broth was flocculated with chitosan 0.06%, calcinated kieselguhr 6%, and activated charcoal 2%, afterwards the fermentation broth pH was adjusted to 5 with HCl 2M, after the method proposed by Wang et al. (2015) [8]. The mixture was stirred at 250 rpm for 30 min at room temperature (23 °C) on a magnetic plate, then vacuum-filtered through MN615 filters, until a clear solution was obtained. The vacuum filtration yield was established using the formula Equation (1).

Filtration yield (%) = volume of clear fermented broth (mL)/volume of fermented broth without biomass (mL) × 100(1)

The clear, fermented broth was concentrated through vacuum distillation for about an hour at 95 °C to remove the water and the ethanol content. The metabolites’ quantity in nonflocculated, flocculated, and concentrated solutions was established by HPLC.

### 2.4. Purification through an Ion Exchange Resin

The filtered solution containing 1,3-PD, 2,3-BD, fractions of glycerol, and organic acids (citric, succinic, lactic, and acetic acids) was passed through an acidic cation exchange resin (Amberlite IR120 H). We filled a glass column of 50-cm height and 3-cm diameter with cationic resin up to 30 cm, to observe the purification yield by using ethanol 75% as eluent. The eluent volume needed to pass through the packed column was calculated using the formula Equation (2).

Veluent = π*r*^2^*h* (2)

where *r*—column radius (or diameter/2) (cm), *h*—column height (cm)

#### Purification Process Using a 30-cm Resin Bed

A 50-cm glass column with a diameter of 3 cm was packed with 30 cm of cationic resin. Before the sample passage through the column, the resin beds were pretreated with 2M HCl, 2M NaOH, and 2M HCl, and then rinsed with deionized water, as mentioned by Wang et al. [8]. Then, 10 mL of filtered broth was passed through the column and eluted with ethanol 75% at a flow rate of 7 mL/min. At regular intervals, 6 mL of effluent was collected to determine the concentration of organic acids, 1,3-PD, 2,3-BD, and glycerol.

**Figure 1:**
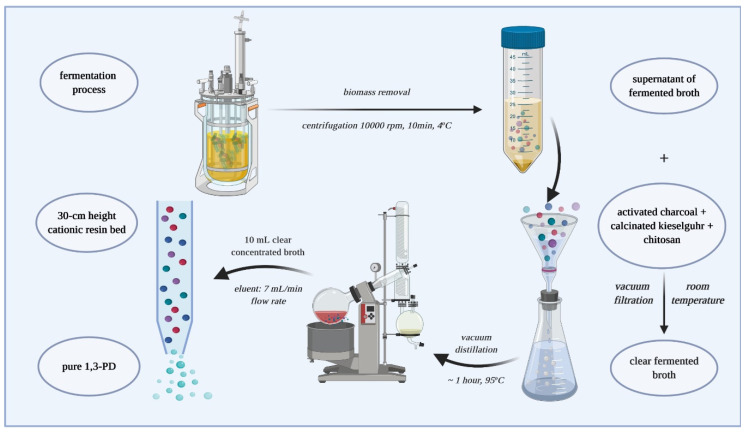
Main steps for obtaining pure 1,3-PD (broth fermentation, biomass separation, flocculation, concentration, purification).

Figure 1 illustrates the main steps for obtaining pure 1,3-PD from glycerol fermentation.

### 2.5. HPLC Analysis

The polyols (1,3-PD, 2,3-BD, and glycerol), the organic acids (citric, succinic, lactic, and acetic acids), and ethanol were identified through HPLC. The system was equipped with a quaternary pump, solvents degasser, and manual injector coupled with a refractive index detector (RID) (Agilent 1200, Santa Clara, CA, USA).

The separation of the compounds was performed on a Polaris Hi-Plex H column, 300 × 7.7 mm (Agilent Technologies, CA, USA), using the 5 mM H_2_SO_4_ mobile phase with a flow rate of 0.6 mL/min, column temperature *T* = 80 °C, and RID temperature *T* = 35 °C. The elution of the compounds was run for 25 min.

The data acquisition and the interpretation of the results were made by using OpenLab CDS-ChemStation Edition software (Agilent Technologies, CA, USA).

## 3. Results and Discussions

The separation and purification of 1,3-PD with high yields is still a challenging process from the technological, environmental, and economic point of view. The industrial production of 1,3-PD utilizing microbes might be negatively influenced, so the purification process of the targeted compound from the microbial fermented media must be optimized and made much more feasible and accessible. In our study, we applied an environment-friendly protocol to separate and purify the interest metabolite from the cultivation broth, namely flocculation, and purification through ion exchange resin.

### 3.1. Broth Flocculation and Concentration

**Figure 2:**
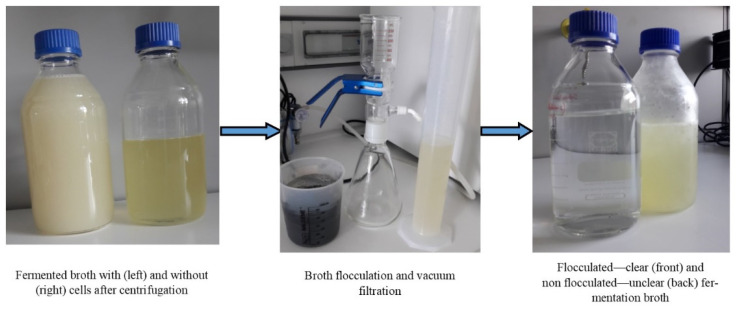
Bacterial cell removal and broth flocculation.

Bacterial cells of *K. pneumoniae* DSMZ 2026 were removed from the culture broth by high-speed centrifugation after 24 h of batch fermentation for 1,3-PD production (Figure 2). The centrifuged culture broth was subjected further to flocculation. The nontoxic flocculation process consisted of chitosan, calcinated kieselguhr, and activated charcoal addition to the cultivation broth, which removed all the colored impurities (proteins, salts, cellular debris) by giving a perfectly transparent solution of biogenic 1,3-PD from a cloudy yellow broth. The vacuum filtration yield was 85%. The vacuum-filtered broth was further concentrated through vacuum distillation and about 265 g/L of a slightly viscous 1,3-PD being obtained as the main metabolite. Hao et al. [14] used flocculation with a mixture of cationic polyelectrolyte chitosan and nonionic polyelectrolyte polyacrylamide for 1,3-PD separation before its purification through reactive extraction with butyraldehyde. Firstly, the authors tested the flocculation with chitosan and polyacrylamide individually, but the broth did not clarify well, especially in the case of broth flocculated with polyacrylamide where it turned more turbid than the original. The authors achieved a recovery ratio of the supernatant liquor to the broth of 99%, the concentration of the metabolites not being mentioned at this step [14].

**Figure 3:**
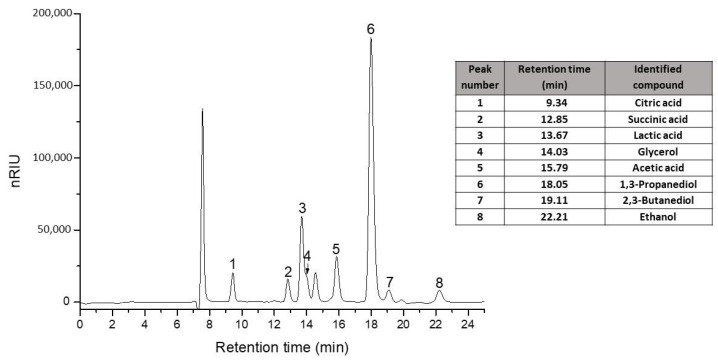
Standard chromatogram and retention time (min) for the interest compounds on a Polaris Hi-Plex H column, Refractive Index (RI) detector.

The cultivation broth was analyzed by HPLC (Figure 3) before flocculation, after flocculation, and after vacuum concentration, and the results are presented in Table 1. In the clear broth, the metabolite concentration slightly decreased towards the cultivation media before flocculation. After almost an hour of vacuum distillation, the quantity of the metabolite was significantly increased.

Compared with other studies, the starting concentration of the metabolites in our study is lower (Table 1). For example, Hao et al. [14] reported 53.5 g/L 1,3-PD, 7.5 g/L 2,3-BD, 27.6 g/L glycerol, 8.1 g/L ethanol, 5.9 g/L lactate, 10.5 g/L acetate, and 5.6 g/L succinate in the fermented broth before being subjected to flocculation [14].

### 3.2. Purification of Biogenic 1,3-PD through a Cationic Exchange Resin

The resin selection was conducted based on a literature review [8,20]. The concentrated, clear broth obtained after biomass removal, flocculation, and vacuum distillation, was separated using the chromatographic purification method [8].

After being treated with acid-base-acid solutions, the 30-cm resin bed was loaded with 10 mL of concentrated clear broth and eluted with a volume of 212 mL ethanol 75%, at a flow rate of 7 mL/min. After each volume of eluent loaded in the packed column, about 6 mL of downloaded samples were withdrawn to establish the eluent volume needed to remove the entire amount of secondary metabolites (organic acids and 2,3-BD) and the unconsumed glycerol during fermentation; 0.05 mL was used for HPLC analysis.

**Figure 4:**
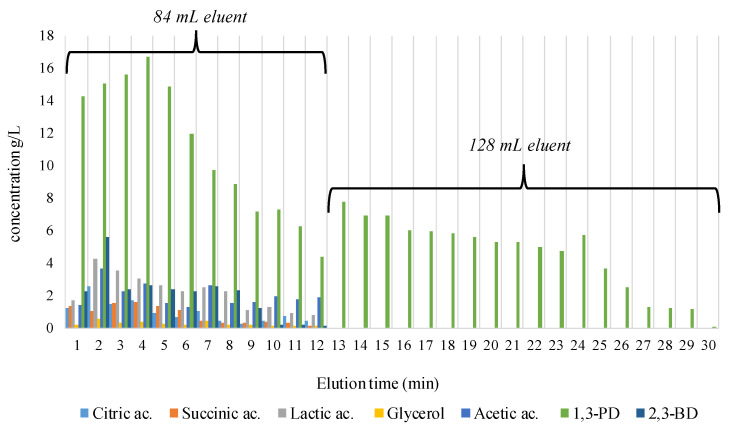
Results obtained for the cationic resin separation of 10 mL of concentrated fermented broth.

We observed that a volume of 84 mL ethanol 75% loaded on the resin column which corresponds to 12 min of elution, completely separated the organic acids, glycerol, and 2,3-BD (Figure 4). An explanation of the clear cut of the components after 12 min of elution may be due to the specific affinity to the stationary/mobile phases. The organic acids, 2,3-BD, and glycerol were flushed earlier mainly because of their elevated affinity to the mobile phase. In the case of 1,3-PD, this has a higher affinity to the stationary phase represented by the column of the resin beds. 1,3-PD creates steric bonds with the cationic resin, an aspect that cumbers the elution process with an increased volume of eluent and longer time to pass through the column being necessary. So, after the column resin loading with the rest of the eluent volume (128 mL) (Figure 4), 80.708 g/L of pure biogenic 1,3-PD, in 18 min of elution was collected. The collected samples consisting of pure 1,3-PD (about 100 mL) were gathered in a glass balloon and concentrated by vacuum distillation at 95 °C until water and ethanol were completely removed. About 15 mL of viscous solution was obtained, with the final content of pure 1,3-PD being 612.03 g/L.

In a study conducted by Leurruk et al [25], between 80 and 160 g/L of pure 1,3-PD was recovered from an aqueous solution of a synthetic mixture by using two cationic exchange resins, Amberlite type XAD-7 and XAD-16. Hao et al. [14] reported that a mixture of metabolites was obtained in a reactive distillation column using the strong acidic cation-exchange resin as a catalyst, specifically 407 g/L 1,3-PD, 252 g/L 2,3-BD, 277 g/L glycerol, and 146 g/L glycerol acetals were obtained [14].

Compared with other techniques used for 1,3-PD separation and purification mentioned in the literature, the method presented in this paper presents some advantages. First, the removal of coarse fragments from the cultivation media implies only centrifugation. Second, the removal of proteins, cellular debris, and other color impurities can be successfully performed by nontoxic flocculation with chitosan, charcoal, and kieselguhr. Third, the removal of organic acids and unconsumed glycerol from the broth can be done by eluting with ethanol a strong acidic resin, like Amberlite IR-120H which can be regenerated periodically. However, there is also a shortcoming of our proposed method considering that an important quantity of 1,3-PD is discharged in the first stage of the purification during the first 12 min of elution. To recover 1,3-PD from this stage, another separation-purification method would be appropriate, like preparative HPLC or another type of resin column.

Regardless, since 1,3-PD has large applicability in the manufacture of biodegradable products such as fibers [35], plastic materials [36], textiles [37], cosmetics [38], food additives [39], or coating materials [40,41], the biogenic 1,3-PD purified through ‘green’ methods and materials represents a feasible alternative to the synthetic 1,3-PD, from an economic and environmental point of view.

## 4. Conclusions

Biogenic 1,3-PD was obtained through glycerol fermentation using *K. pneumoniae* DSMZ 2026 as a bioconverter. The cultivation media consisted of 1,3-PD, 2,3-BD, organic acids (lactic, citric, succinic, and acetic acids), and ethanol. The main steps to obtain pure biogenic 1,3-PD from the cultivation broth were separation through flocculation with a mixture of chitosan, activated charcoal, and kieselguhr, and purification through a cation exchange resin H^+^ form. It was observed that cell debris, pigments, and protein fragments were completely removed through flocculation resulting in a perfectly transparent, aqueous solution of metabolites, with low differences between their concentrations before and after the process. The concentrated broth—10 mL—was loaded in a 30-cm height resin column, and a volume of 84 mL of ethanol 75% as eluent loaded with a flow rate of 7 mL/min was necessary to entirely remove the secondary metabolites (organic acids and 2,3-BD) and the residual glycerol, during 12 min of eluation. Pure biogenic 1,3-PD was recovered in 128 mL of the eluent, which, after being subjected to vacuum concentration (7 times concentrated), yielded 91% of biogenic 1,3-PD in its pure form.

In the light of the scope of our research, the results presented in this paper make the separation and purification method valuable considering the environmental and economic aspects, as the materials used are biodegradable and renewable. The biogenic 1,3-PD obtained through the microbial conversion of glycerol and purified through reusable ionic resin represents a feasible alternative to the synthetic production of 1,3-PD, and at the same time a valuable alternative that would answer to the increased demands of 1,3-PD on the market.

## Figures and Tables

**Table 1:** Metabolites and unconsumed glycerol concentration (g/L) in the fermentation broth (before/after flocculation and after concentration).

Medium/Compounds Header	Citric Ac.	Succinic Ac.	Lactic Ac.	Glycerol	Acetic Ac.	1,3-PD	2,3-BD	Ethanol
Nonflocculated Fermentation Broth	1.62	1.17	3.42	0.30	4.06	28.86	4.89	1.43
Flocculated (Clear) Fermentation Broth	1.31	0.97	2.83	0.28	3.28	26.88	2.93	1.23
Concentrated Fermentation Broth	12.52	9.22	30.01	3.52	27.56	265.06	33.31	-
